# Gynecological Society

**Published:** 1886-02

**Authors:** 

**Affiliations:** 2330 Indiana Avenue


### SOCIETY REPORTS.

---

TRANSACTIONS OF THE GYNÆCOLOGICAL SOCIETY OF CHICAGO.

*Regular Meeting, 18th December, 1885.*

I. JAGGARD. *Two Recent Models of the Axis-Traction Forceps.*

II. BYFORD. *Report of a Case of Pelvic Abscess, with Remarks upon the Treatment.*

The PRESIDENT, DANIEL T. NELSON, M. D., in the chair.

I. PROFESSOR W. W. JAGGARD read a paper entitled, TWO RECENT MODELS OF THE AXIS-TRACTION FORCEPS.

The object of the paper was not the description of some modification by the writer, although such a contribution to the literature of the subject would be perfectly legitimate in view of Pajot's witty remark to the effect "that he does not reproach a man for having invented a forceps, since that might happen to any one."—(*Barnes.*)

BREUS and FELSENREICH, formerly assistants respectively in the third and first obstetrical clinics of the Vienna General Hospital, have recently made important alterations of Tarnier's axis-traction forceps. The importance of these modifications was so great that no apology was demanded for calling attention to the instruments.

*Historical.*—As the result of the labors of Sir J. Y. Simpson, Nägele, Busch, Levret and others, the low forceps operation may be regarded as a comparatively perfect operative procedure, both as regards instruments and mode of operation. The case is different with the high forceps operation. This operation is always difficult, and sometimes dangerous, with the instruments mentioned. The cause is obvious. The applied force can be resolved into two components, one in the direction of the axis of the plane of the inlet, the other perpendicular to the first, directed towards the posterior surface of the symphysis. The first component is alone active in causing the descent of the head; the second makes the extraction more difficult and exposes the maternal tissues between the head and symphysis to traumatism. As remarked by Schauta, (*Grundriss der Operativen Geburtshilfe*, Wien, 1885, p. 162,) "the unphysiological and therefore mischievous element in the operation of the forceps, as compared with the effects of uterine contractions, when the head is at the inlet, consists in the fact that the forceps draws the firmly-held head in a direction which it can never follow, while the uterine contractions simply drive the head into the pelvic cavity, and permit it after that to seek the direction of least resistance." The older obstetricians, fully recognizing these facts, attempted to apply the power to the classical forceps, in such a way as to secure a more favorable direction of traction. Osiander (1799) and Stein, Sr. (1805) may be mentioned among the older obstetricians, who devised instruments for making traction in the axis of the inlet. Hermann (1844) (Kilian's *Armamentarium lucinæ novum*) constructed an instrument, in which an iron lever is attached to the lock. J. P. Hubert (1860) attached a vertical iron lever to the extremities of the ordinary forceps. This lever was subsequently attached to the lock. Eugéne Hubert, his son, constructed an axis-traction forceps, with parallel branches and a sharp perineal curve. Chassagny, Joulin, Pros, Poullet, Moralès Apaca (1871) and others have constructed various types of axis-traction forceps at a more recent period. In many of the modern French instruments an attempt has been made to apply some of the well-known principles of veterinary surgery.

TARNIER'S FORCEPS. In 1877, Tarnier, following in the wake of Hermann, Hubert, and the more recent French inventors, constructed and published a description of his well-known instrument. Since that time, he has produced more than thirty distinct models. His last model consists of the classical forceps of Levret, (without a perineal curve) and axis-traction rods attached to the posterior, inferior border of the blades, or spoons. Tarnier claims a number of advantages for his instrument over any other axis-traction forceps. He claims that it is superior to the classical instruments in the following particulars:

1. It is possible to apply traction in the direction of the principal pelvic axis.

2. Sufficient mobility is conferred upon the child's head to permit it to seek its way through the pelvis in the direction of the least resistance.

3. The handles indicate to the operator the direction in which traction should be made.

With reference to the first proposition, it may be said that traction with Tarnier's forceps is not made in a curved line, accurately coincident with the principal pelvic axis, when the head is at the inlet. Nor is traction in this direction absolutely necessary, as remarked by Schauta, seeing that the resultant of the forces, developed by uterine contractions, and the resistance opposed by the pelvic floor, does not propel the head in the direction of the principal pelvic axis.

The handles, as indicators of the direction in which traction should be made, are of relatively slight value.

On the one hand, the operator who is at all qualified to apply the forceps to the head at the inlet, ought to have a correct conception of the direction in which traction should be made. On the other hand, strict attention to the handles may prevent the operator from observing a number of important events, ex. gr., the relation of the head to the vulva, slipping of the instrument, etc. (Schauta.)

Finally, the handles are not a correct indicator of the direction of the principal pelvic axis.

The advantage of Tarnier's forceps over its predecessors lies in the mobility conferred upon the fœtal head by the joint, uniting the blades and the so-called axis-traction rods. The head does not follow the direction of the principal pelvic axis, but seeks the path of least resistance. In consequence, the operator is spared the fatigue of unnecessary effort, and the mother, the dangers of traumatism from violent traction.

I. BREUS has recently constructed an instrument which has a great advantage over the forceps of Tarnier, in that a greater degree of mobility, during traction, is conferred upon the head.

The continuity of the blades (*Löffel*) is interrupted at, and below, the fenestræ, by a strong flat joint, which admits of movements in the sagittal direction, and corresponding variability in the angle at which traction is applied to the head. The superior ribs of the instrument are prolonged, and turned upward like spurs. These spur-like prolongations are joined by a metallic rod in order to preserve a certain parallelism of the blades.

Apart from these peculiarities, the instrument is identical with the original model of Sir James Y. Simpson's forceps.

This instrument, devised by an obstetrician of large experience, is employed on an extensive scale at Vienna, in Gustav Braun's obstetrical clinic. Schauta (*Grundriss der Operativen Geburtshilfe*, Wien, 1885, p. 164 et seq.) recommends the instrument as the most perfect axis-traction forceps in existence, to his classes at the University of Innsbruck. Fürst's recent favorable note on Breus's forceps in the *Centralblatt für Gynækologie*, 1885, is well known.

II. FELSENREICH'S MODIFICATION OF PROFESSOR ALEXANDER SIMPSON'S MODIFICATION OF TARNIER'S AXIS-TRACTION FORCEPS.

In 1880, Professor Alexander Simpson, of Edinburgh, sent to Professor Carl Braun a modification of Tarnier's axis-traction forceps, which at once superseded the French instrument in the first obstetrical clinic of the Vienna General Hospital. Simpson substituted Sir J. Y. Simpson's original model of the classical instrument for Levret's. The compression-screw is located on the upper third of the superior surface of the handles. Comparatively unimportant modifications were made with reference to the traction-rods, and the hard-rubber handle, into which the traction-rods fit. Felsenreich has materially enhanced the value of Professor Alexander Simpson's instrument by a number of important alterations.

Felsenreich's modification of Professor Alexander Simpson's axis-traction forceps, as shown by the model presented, manufactured by Mr. J. Leiter, of Vienna, during October, 1885, consists of the following parts:

I. A practically unaltered model of Sir James Y. Simpson's forceps (*Wiener Schulzange*).

II. Button-hole perforations, one behind each fenestra, into which traction-rods are inserted, and maintained by the buttons on the ends of the rods.

III. A removable compression thumb-screw, which sinks into a groove made in the extremities of the handles of the Simpson forceps.

IV. A hard-rubber handle for the traction-rods. The arrangement for the insertion of the traction-rods into the hard-rubber handles differs from the mechanisms in Tarnier's and Alexander Simpson's axis-traction forceps.

The attachment of the compression-screw to the ends of the handles, and certain changes in the curve of the axis-traction rods have been made at a comparatively recent period, but prior to 1883.

Dr. L. E. Neale, of Baltimore, published an article in the September number of the *American Journal of Obstetrics*, 1885, entitled "An Obstetric Forceps." In this paper Dr. Neale describes an axis-traction forceps, devised by himself, which differs in no essential particular from Felsenreich's modification of Alexander Simpson's instrument. Editorials have appeared in the *Journal of the American Medical Association*, 26th September, 1885, and the *Therapeutic Gazette*, 15th December, 1885, calling attention to the facts, that a forceps, identical with the instrument devised by Dr. Neale in all essential details, had been constructed several years before in Vienna by Dr. Felsenreich, and that Dr. Neale had probably forgotten the existence of that instrument, although he had seen it in active operation in the lying-in ward of Carl Braun, in various courses on operative obstetrics, and in the shop of Mr. J. Leiter, the instrument-maker to the Vienna General Hospital. The only criticism that the writer would make, with reference to these editorial notes, was, that Dr. Felsenreich, not Dr. Neale, applied the compression thumb-screw to the ends of the handles. Dr. Neale has made some trivial modifications in the hard-rubber handle and the mode of insertion of the traction-rods. Dr. Neale made no allusion to Alexander Simpson's modification of Tarnier's instrument in the paper mentioned, and his allusion to Dr. Felsenreich's suggestion of the button-hole joint is, to put the case very mildly, disingenuous.

In conclusion, Professor Jaggard said that he was of the opinion that the axis-traction forceps of Breus and Felsenreich were superior to the most recent model of Tarnier's, or any other axis-traction forceps that had come under his observation. He requested that the discussion be limited to the comparative merits of the forceps presented—Breus's and Felsenreich's—and other recent models of the axis-traction instrument.

DISCUSSION.

DR. JOHN BARTLETT said that he had devised an axis-traction forceps in 1880, identical in principle with the instrument constructed in 1860 by the elder Hubert. His attention had been first called to the coincidence by Professor Lahs's monograph on *Die Achsenzug-Zangen*, Stuttgart, 1881.

DR. HENRY T. BYFORD thought the instrument described by Dr. Neale in the September number of the *American Journal of Obstetrics*, 1885, was identical in all essential particulars with

Felsenreich's modification of Alexander Simpson's instrument devised two or three years before.

DR. PHILIP ADOLPHUS, PROFESSOR A. REEVES JACKSON, DR. H. P. MERRIMAN, DR. H. P. NEWMAN had never observed indications for axis-traction forceps; had never employed such instruments, and thought they were unnecessary.

PROFESSOR JAGGARD said he had no desire or intention to discuss the general subject of axis-traction forceps, and had expressly requested that the discussion should be limited to the consideration of the relative merits of the instruments presented for examination, (Breus' and Felsenreich's) and other modifications of the axis-traction forceps. He thought that gentlemen of limited experience in cases indicating the high forceps operation, and particularly those who had absolutely no experience with axis-traction instruments, should be temperate in their criticism. Carl Braun, Pajot, Charpentier and others had practically rejected such instruments, but only after serious and experimental consideration of their merits. On the other hand, many younger obstetricians, including Schauta, Felsenreich, Breus, Ehrendorfer, thought there were cases in which they might be profitably employed.

II. DR. HENRY T. BYFORD read a paper, entitled, REPORT OF A CASE OF PELVIC ABSCESS, WITH REMARKS UPON THE TREATMENT.

Mrs. T., aged twenty-five years; married five years; German descent; of nervous temperament; small and slight in figure, but in good general health, consulted me, during the fall of the year 1884, for sterility and dysmenorrhœa. She had never menstruated without pain, but had otherwise enjoyed good health. An examination revealed a small uterus and cervix, with acute anteflexion and consequent apposition of the anterior and posterior uterine walls. Slippery elm tents, used about once in eight days, alternated with glycerine tampons, had for their effect a gradual relief of the dysmenorrhœa.

About the middle of the following February, I was called to her house to treat her for a severe attack of pelvic cellulitis, contracted a week before while returning home from a dance. The whole pelvic connective-tissue seemed involved, and large tender lumps could be felt externally in the left iliac region.

Six weeks from the beginning of the attack, an abscess opened into the anterior wall of the rectum, about two inches from the external anal orifice. On account of the extreme debility of the patient, her horror of operative procedures, and the absence of any well-marked fluctuation, all surgical interference with the suppurative process had been out of the question.

Palliative treatment was instituted and continued without effect until the sixth of June. In the meantime the pulse remained in the neighborhood of 120° F., and the temperature fluctuated between 99° F. and 102° F.; attacks of acute suffering and septicæmic diarrhœa required opiates for their relief; the bacillus tuberculosis was discovered in the pus; yellow pigmentary deposits covered her face, and emaciation became extreme, her weight ranging between eighty-two and eighty-three and one-half pounds. Her courage began to fail, and finally after the concurrent recommendation of the consultants, Drs. Wm. H. Byford, J. E. Owens, George M. Chamberlin and Martin Matter, she consented to an operation.

Accordingly, on the 6th of June, Dr. Wm. H. Byford operated according to his usual method in such cases. After etherization, he forcibly dilated the sphincter of the anus, tore open the fistulous track with the finger, and then enlarged the abscess in the same manner, in the direction of the lowest part of the cavity, until it readily admitted two fingers. I then made a digital examination, and found the abscess to extend across the pelvis, behind the uterus and broad ligaments, above the level of the fundus uteri on the left side, and to be filled with bands and projecting masses of granulation-tissue of about the consistency of freshly coagulated blood. Previous treatment, except to diminish and control the septicæmia, had evidently been a complete failure All of this medullary tissue was then scooped out with the finger and the cavity thoroughly cleansed with a two and a half per cent. solution of carbolic acid.

The highest temperature after the operation was 99° F., on the day following. Perfect drainage had been secured, for at the time of each dressing no pus was found inside of the abscess.

The cavity of the abscess was treated by irrigation with antiseptic solutions, insufflation with iodoform and the introduction of cupric sulphate.

Early in September she was attacked with the then prevalent epidemic, dysentery, and died on the 23d instant.

At the post-mortem examination made about thirty hours after death, I was somewhat hampered on account of a promise, exacted by the husband, that no organ should be taken out of the body, and by the fact that I had but thirty minutes for work before train time. The body had again become extremely emaciated. Abdomen was flat. An incision was made from a little above the umbilicus to the pubic bone. The pelvis was filled posteriorly with a solid mass of plastic tissue, which had drawn the uterus backwards to within about half an inch of the sacrum, so as to put the anterior vaginal wall upon the stretch, and had buried the uterus and other pelvic organs in its substance. Both round ligaments were seen issuing from this mass. It was necessary to cut down about half an inch before reaching the depressed uterus, and to tear through solid tissue behind it to arrive at the rectum below. The finger broke through into the rectum, behind the dimpled cicatrix that marked the site of the former outlet of the abscess. The left broad ligament was then felt to be represented by, or inclosed in, a tough band half an inch thick antero-posteriorly, extending from the uterus to the left side of the pelvis. The left ovary could not be found. A small flat piece of what seemed to be ovarian tissue was found adherent to the bladder on the right side. The right broad ligament was apparently disorganized and inseparable from the plastic deposit. The rectum was held inflated at the point where it issued from the pelvis, was dark colored and injected on its external surface, and blackish and softened on the internal. Neither the appearance nor the odor of an abscess could anywhere be discovered.

There seem to have been two hinges, as it were, upon which the treatment of this abscess turned: first, the operation per rectum: second, the cauterization by sulphate of copper. Both secured a large opening at the lowest portion of the pyogenic cavity, and brought away the unhealthy granulation-tissue. Had the patient consented to have the unobstructed outflow of the pus maintained by one or two subsequent dilatations, similar to the first one, the cure would undoubtedly have been more rapid. As it was, the contracting sphincter and abscess outlet rendered the drainage and irrigation imperfect. Progress toward recovery was, however, again inaugurated upon the melting away by the sulphate of copper of the newly and imperfectly formed cicatrical tissue, reproducing the opening made at the time of the operation and by the destruction of the degenerative deposits and cauterization of the chronic pyogenic surface. The only kind of treatment preferable to this free drainage and clearing out method is the strictly antiseptic, which, after the pus has once found a way into the rectum, can only be accomplished by first closing this septic inlet.

The treatment by a counter opening in the vagina is much less preferable, because a recto-vaginal fistula, difficult of cure, and liable, like anal fistula, to inoculate the system with tuberculosis, would be left.

The treatment by abdominal incision cannot for a moment be entertained, for at least two reasons:

1st. It is necessarily followed by a recto-abdominal fistula of great length, which is incapable of being promptly cured, and is apt to become an unfailing source of systemic infection. Those patients already operated upon, as far as reported, have usually either died shortly, or within a year or two, imperfectly cured. They would have, on an average, lived about as long without the operation. In fact, it is not impossible that one such, whom I had, previous to the operation, an opportunity of watching for a short time, would finally have recovered through the process of nature. To operate as does Lawson Tait, before the abscess has discharged, and then treat it antiseptically through its single opening, is an entirely different matter.

2d. The danger of an abdominal incision should never be incurred without a prospect of compensation in the way of bettering the patient's chances of recovery. Neither theory nor practice as yet prove such compensation to be attainable.

In some cases one dilatation per rectum, without after-treatment, has sufficed for a cure; in other cases two or more, with subsequent antiseptic irrigations, have become necessary. But as a general rule it may be said that, unless instituted too late, the procedure is safe and the recovery sure.

DISCUSSION.

PROFESSOR CHRISTIAN FENGER made some remarks on laparotomy as compared with other operations, of which the following is a brief abstract:

When a peri-uterine abscess points somewhere in the vagina around the lower part of the uterus, no surgeon would, of course, think of doing anything but opening the abscess, inserting a drainage tube, and by washing out, endeavoring to effect the closure of the cavity. But in some cases the opening into the vagina is just as ineffective as a spontaneous opening into the rectum. In obstinate cases of this kind laparotomy, at a later period, will have to be performed.

There is, however, no doubt that secondary invasion of septic poison, when the abscess is opened from the vagina, is much more difficult to prevent than invasion into the abscess from the abdominal opening. It is only in this way that we can account for the difference in the course of the after-treatment of peri-uterine abscesses opened through the vagina and through the abdominal cavity; a difference that Lawson Tait rightly calls attention to as being decidedly in favor of the abdominal operation. Here the abscess closes more quickly, and the course of the after-treatment is much less febrile than in the vaginal operation.

Sometimes a peri-uterine abscess will point into the rectum, sufficiently low down to permit of an opening here. It does not seem probable that the access from the rectum will be very promising, as effective drainage is next to impossible; but the cases of cure by spontaneous opening into the rectum evidently make an operation here permissible, and perhaps advisable, but only as a trial. If the abscess does not retract within a reasonable time, other measures must be resorted to.

It is needless to state that if a parametritic abscess points anywhere along the iliac fossa, it should be opened and drained from this point; but this does not belong to my subject of to-night, as I desire to call attention only to strictly circum-uterine abscesses, which can only be reached from the vagina or from the supra-pubic region.

When a circum-uterine abscess does not point downward, and, in fact, does not point anywhere, it is then the surgeon's task to find the safest way into the abscess through a smaller or larger amount of surrounding tissues.

We shall first consider the vaginal operation:

When so eminent an authority as Schröder, of Berlin, advocates this method of reaching a high peri-uterine abscess there must be cases in which this operation is advisable. From a general point of view an extra-peritoneal outlet of the abscess through the vagina would seem to be safer than laparotomy, upon the same grounds as a vaginal hysterectomy is safer than Freund's abdominal hysterectomy, and Schröder's successful operation, already mentioned, vouches for the method.

At the same time, I firmly agree with Lawson Tait, that there are some grave objections to the vaginal operation. In the first place, a high-seated peri-uterine abscess is difficult to reach. It is difficult to work with safety two or three inches above the introitus of the vagina, in tissues that are immovable, and where the parts cannot be drawn down toward the operator. These difficulties are, of course, of less importance in the master hands of an operator like Schröder, but increase in significance for less experienced surgeons.

But the operation through the vagina is more or less an operation in the dark. We may be dissecting up along the posterior surface of the neck of the uterus, and may open into recesses of the peritoneal cavity between the abscess and the uterus. Further, it might be easy in this place to open into the rectum.

Another danger, especially in abscesses between the two layers of the lateral ligament, might easily arise from the rupture of the large uterine vessels running in the wall of the sac. It would be exceedingly difficult, and I should say next to impossible, under such circumstances, to secure and ligate these vessels, the point of ligation being so high up, the working space so small, and the tissues so immovable.

All those objections and dangers we do not encounter in laparotomy. We can see distinctly, and recognize with our own eyes, every particle of tissue we have to divide; the large uterine vessels, if divided, can easily be taken up and ligated. There is no risk of having any communication between the abscess and the peritoneal cavity, which we cannot either close up or drain.

If the laparotomy lasts longer, and gives more technical work to the surgeon, it seems to me that these objections are fully balanced by the advantage of not being obliged to operate in the dark, of not having to battle with enemies that we cannot see, and consequently cannot guard against.

But these are not the only advantages of laparotomy, as compared with the vaginal operation. The free access to the whole interior of the abscess cavity has also to be taken into account. By laparotomy, the abscess is laid open to about the same extent as a tubercular peri-articular abscess. We can examine the whole interior of such a cavity, and scrape off, or remove by other means, whatever objectionable material we may find, cheesy matter, tuberculous tissue, fungoid granulations—since we can see clearly every place where the instrument is applied, without any danger of going through the abscess wall into any surrounding cavity or organ.

It is more than possible that this free access to the abscess wall has something to do with the speedy recovery subsequent to laparotomy, as compared with the vaginal operation.*

But, of course, there will always be connected with laparotomy the inherited dread of opening that ominous peritoneal cavity. Modern surgery, however, is making steady progress in diminishing these dangers. Thus, the dread, as well as the safety of the patient, will, to a great extent, rest in, or depend upon, the care and skill of the operator.

PROFESSOR W. H. BYFORD: I do not wish to comment upon the contents of the paper further than to express myself in reference to the mode of operating adopted in consultation with the gentlemen mentioned. A large number of pelvic abscesses can be managed through the rectum with more facility and safety, than any other medium of approach to the deep-seated portions of the pelvic cavity. I do not know whether there are any cases wholly situated in the pelvic cavity, but that can be reached, opened and evacuated through the rectum. It may not always be the most eligible direction to approach collections of pus. In instances in which the pus is making its way toward the vagina, and fluctuation can be felt through the vaginal walls, it ought to be evacuated through that canal; but when the point of discharge is not thus indicated the exploration is most easily made through the rectum; and all chronic cases that have already commenced to discharge into the rectum can and ought to be treated from the cavity of that viscus. I would make no exception, however high the opening might be, so it was within the pelvic cavity. By proper preparation the whole length of the rectum can be reached

* Lawson Tait, op. cit.

from the sphincter to the promontory of the sacrum, and from any part of it the pus evacuated; the pyogenic cavity explored and drainage and irrigation safely and securely accomplished.

I believe the dangers of this mode of operating to be incomparably less than by abdominal section; and the other results of the operation—such as drainage and disinfection—more complete.

To effect the objects mentioned, the sphincter should be stretched to laceration; and until there is no tendency to immediate contractions of the anal opening, and till it can be dilated to the full extent of the rectal cavity. Thus thoroughly opened, the whole extent of the rectum can be explored with great facility and often by means of dilators can be seen, and instruments used under the eye of the operator.

If the pus is to be sought after, palpation with the fingers becomes easy and satisfactory; if it is being evacuated, the orifice seen or felt and such treatment as is desired applied. I very much prefer stretching and tearing for the purpose of increasing the size of the discharging orifice to the use of cutting instruments. The opening will not so readily close and there will not be so much hæmorrhage.

In effecting the discharge of the pus, we should remember that the reason why the pyogenic cavity is at no time wholly obliterated is because there are irregular loculi or pockets so situated that they do not empty themselves. The opening should therefore be made large; the parts torn by the fingers until this inferior margin of the opening is as far below the main body of the cavity as practicable. With the fingers the interior bands and partitions should be completely broken down and the interior of the cavity rendered as nearly symmetrical as possible. This will enable the whole of the contents of the cavity to escape by means of gravity, and the fluids used in irrigation find their way out without difficulty. In addition to this shaping of the cavity, the large granulations—generally so abundant—should be scraped away by the fingers or by a dull curette, thus freshening up the lining membrane of the pyogenic cavity and converting it from a state of indolent ulceration to one disposed to heal. This process of curetting also produces a change in the capillary circulation that makes nutritive processes more salutary. Often in very indolent cases the sphincter will recover contractile power to such a degree as to require one or more repetitions of the operation. The same thing may be said of the margin of the orifice in the intestine. We will be obliged to enlarge it and treat the cavity as before.

In the case narrated in the paper, the action of the sulphate of copper seemed most useful and contributed the last influence necessary to the cure.

I have said nothing about the more common items of treatment, such as irrigation, disinfection and stimulation. My intention is to show the facility with which, in many instances, these purulent collections can be reached and treated by dilating and distending the rectum and the comparative safety of such proceedings.

PROFESSOR E. C. DUDLEY: The experience of Dr. Byford and others in the treatment of pelvic abscess by this operation must be considered as proving the great value of the operation in cases in which the abscess can be easily approached and thoroughly drained by dilatation of a sinus between the abscess-cavity and the rectum. It would, however, appear on general principles, that sufficiently free and long-continued drainage would in many cases be almost unattainable and that an abscess-cavity left thus to heal must often be the starting point of sinuses formed by the uncontrolled burrowing of pus in many directions. The almost inevitable invasion of the abscess-cavity by fecal matter is clearly a serious factor in connection with the history of these cases. The great mortality from pelvic abscesses opening spontaneously into the bowel demonstrates the inability of nature to provide for adequate drainage. Whatever question, therefore, we may raise relative to the advanced position of Dr. Wm. H. Byford, who, if practicable, would prefer to open a pelvic abscess through the rectum—even in those cases in which nature has not anticipated him—there can be no question about the propriety of enlarging and rendering more effective an opening already formed. I regret that the essayist has marred a most admirable contribution by the sweeping statement that in all cases in which drainage has been spontaneously established through the rectum Lawson Tait's operation is contra-indicated. Nor can I imagine from what premises he has formed the conclusion that Tait's operation prevents closure of the sinus between the abscess-cavity and the rectum. The question naturally arises whether Tait's operation might not in such cases fulfil a well recognized surgical indication by establishing a free counter-opening for an abscess which otherwise might refuse to close on account of imperfect drainage and on account of its forming a blind sac for the retention of fecal matter. To a larger number of recognized authorities, who deem an opening into the rectum, whether produced by nature or by art, a grave misfortune, the query would naturally arise whether such an opening ought not to be supplemented by a counter-opening, which would bring the draining and cleansing of the abscess-cavity within the easy and absolute control of the surgeon. Furthermore, in view of the decided mortality which attends the spontaneous opening of pelvic abscesses into the rectum, and in view of the almost uniformly successful results recorded in the statistics of Tait's operation already published by Mr. Tait and others, and in view of a very generally accepted rule that the operator in opening a pelvic abscess should strive to keep out of the rectum, I don't think a statement that the rectum is to be preferred as the site of the primary operation ought to go on the records of this society unchallenged.

PROFESSOR J. T. JELKS (present by invitation) thought a great mistake was made in waiting too long before operating in cases of chronic pelvic abscess.

DR. PHILIP ADOLPHUS thought the paper was beyond the pale of criticism. When the general symptoms indicated a collection of pus, the cavity should be searched for. If a cavity containing serum was found, an operation was contraindicated. If the cavity contained pus, it should be evacuated.

In closing the discussion, DR. H. T. BYFORD objected to the quotation of Lawson Tait's statistical triumphs in this connection. In the last edition of Tait's *Diseases of the Ovaries*, abdominal section is recommended for those pelvic abscesses only that cannot be successfully evacuated from below. They are generally such as are situated high up, and do not point early in the vagina or rectum, or they are suppurating hæmatoceles.

The statement that the recto-abdominal fistula, left after abdominal section for a pelvic abscess that has already discharged into the rectum, would heal readily, like any artificial anus, is not borne out by facts. Fistulæ connecting the rectum with the external air have seldom healed, when left to themselves, before a long period of time had elapsed. Operative measures cannot (in these cases) be resorted to on account of the length, situation and relations of the fistulous track.

W. W. JAGGARD, M.D.,
*Editor.*

2330 Indiana Avenue,
25th Jan., 1886.

